# Changes in echo intensity of the gastrocnemius muscle with passive ankle dorsiflexion: can echo intensity be used to assess muscle elongation?

**DOI:** 10.3389/fphys.2023.1197503

**Published:** 2023-09-15

**Authors:** Sayaka Nakao, Tome Ikezoe, Masahide Yagi, Jun Umehara, Shusuke Nojiri, Noriaki Ichihashi

**Affiliations:** ^1^ Human Health Sciences, Graduate School of Medicine, Kyoto University, Kyoto, Japan; ^2^ Faculty of Rehabilitation, Kansai Medical University, Osaka, Japan

**Keywords:** echogenicity, echo intensity, gray scale, shear elastic modulus, shear modulus, stretching, ultrasound, ultrasonography

## Abstract

**Introduction:** While shear modulus has been used as an index of muscle elongation, high costs prevent its general adoption. A more general indicator that can quantify muscle elongation non-invasively is needed to develop effective methods for stretching each muscle. The purpose of this study was to determine whether the echo intensity of the muscle changes with muscle elongation compared with the shear modulus.

**Methods:** Sixteen healthy males (24.1 ± 2.8 years) participated in the study. Shear modulus and echo intensity of the medial gastrocnemius were assessed at 20° and 10° of ankle plantar-flexion, and 0°, 10°, and 20° of ankle dorsiflexion (presented as −20°, −10°, and 0°, +10°, +20°, respectively). Shear modulus was measured using ultrasound shear wave elastography. The echo intensity was quantified using the average grayscale value of a given region of interest (RoI) in longitudinal and transverse B-mode images. Grayscale analysis was performed using two RoIs: one which included as much of the muscle as possible (maximum RoI), and a rectangular one whose size and depth was identical for all images (rectangular RoI). Pearson’s correlation coefficients between either the shear modulus or echo intensity and the ankle angle and between the echo intensity and shear modulus were calculated separately for each participant.

**Results:** Average Pearson’s correlation coefficient between shear modulus and ankle angle of the participants was 0.904. The average Pearson’s correlation coefficients between the echo intensity and ankle angle were estimated to be 0.797 and 0.222 for the maximum RoI and 0.698 and 0.323 for the rectangular RoI in the longitudinal and transverse images, respectively. The average Pearson’s correlation coefficients between the echo intensity and shear modulus were 0.684 and 0.514 for the maximum RoI, and 0.611 and 0.409 for rectangular RoI in the longitudinal and transverse images, respectively.

**Discussion:** The results indicate that the echo intensity in the longitudinal image of the gastrocnemius, especially when assessed using the maximum RoI, increased with muscle elongation by passive ankle dorsiflexion. Therefore, assessment of the echo intensity using the maximum RoI in the longitudinal image might be useful for quantifying the muscle elongation.

## 1 Introduction

An indicator that can quantify the elongation of individual muscle non-invasively is needed in order to develop effective methods for stretching each muscle. Shear modulus, which is measured using the ultrasound shear wave elastography, has recently been used to assess the muscle elongation (Umehara et al., 2021; Mukai et al., 2022); however, the high cost of this device prevents its adaptation for clinical or research institutions. A previous study assessed muscle elongation using ultrasonography and real-time motion analysis; however, this form of data processing is complicated because it requires analysis of the distance between reflective markers and subsequent calculation (Kay et al., 2015). Therefore, it is much needed to identify a more general indicator that can assess muscle elongation non-invasively with simple data processing.

Echo intensity (EI) is quantified using the average grayscale value of the given region of interest (RoI) in an ultrasound B-mode image, which ranges from 0 [black] to 255 [white]. The B-mode image displays white in the portion where the reflected ultrasound wave is high, and this corresponds to the high EI. The ultrasound wave gets reflected on the fibrous connective tissue, such as vessels, perimysium, and epimysium (Pillen and van Alfen, 2011; Mayans et al., 2012). The amplitude of the reflected wave increases when the ultrasound wave enters perpendicularly to the tissue (Madaras et al., 1988). A recent study showed that EI increased when the fascicle angle decreased (Pinto et al., 2023). Therefore, it is hypothesized that when a pennation angle decreases upon stretching a muscle (Abellaneda et al., 2009), the amplitude of a reflected wave on perimysium increase, thereby resulting in the high EI. Furthermore, perimysium stretching, and reorientation of muscle fascicles may also change the EI. When a muscle is elongated, the slackened muscle fascicles would rearrange three-dimensionally (Takahashi et al., 2023), and the number or range of muscle fascicles included in the B-mode image may change. If the reflective planes of the ultrasound wave increase, the EI increases. If the muscle EI increases with passive muscle stretching, EI could be used as a more general and useful indicator to assess the muscle elongation non-invasively.

The effective methodology for using EI to quantify muscle elongation is unclear, since the change in EI depends on how the scan and analysis of the ultrasound B-mode image are performed. First, for the scan setting, the EI values would change depending on whether a B-mode image is scanned transversely or longitudinally to the muscle. The transversely-scanned images are often used when assessing the muscle quality (Arts et al., 2010; Fukumoto et al., 2012). However, longitudinally-scanned image, which capture the muscle fascicles more widely, might be preferred than the transversely-scanned image in order to investigate the changes in EI due to the changes in orientations of muscle fibers with muscle elongation. Second, for analyzing the images after the scan, grayscale analysis for quantifying EI has been established mainly by using two types of RoIs: one which included as much of the muscle as possible (maximum RoI), and a rectangular one whose size and depth were identical for all images (rectangular RoI) (Caresio et al., 2015). Rectangular RoI might be more suitable as the depth affects the EI values because the ultrasound wave decays with deeper depths and the amplitude of a reflected wave decreases. Conversely, the rectangular RoI has a disadvantage due to its limited RoI within the muscle, which is because the size and location of the RoI is determined by using the image in which the muscle thickness is smallest among all the images. Therefore, it is important to clarify the effective scanning and analysis protocols to assess the changes in EI with passive muscle elongation, which will be used as an indicator of muscle elongation.

In this study, we aimed to investigate whether the EI of the gastrocnemius muscle changes with muscle elongation by passive ankle dorsiflexion when compared with the shear modulus. It was hypothesized that EI, and not just the shear modulus, of the gastrocnemius muscle would increase with passive ankle dorsiflexion. We also aimed to investigate the effective scanning and analysis methods to assess the changes in EI depending on the angle of the ankle joint. Specifically, we investigated which probe orientation is preferred for the scanning setting (i.e., transverse or longitudinal to the muscle), and which RoI setting is more suitable to quantify EI using the grayscale analysis (i.e., maximum RoI or rectangular RoI). We hypothesized that more changes in EI would be observed with muscle elongation by passive ankle dorsiflexion, particularly in the EI assessed using the longitudinal image and maximum RoI, as opposed to the transverse image or rectangular RoI. This is because the longitudinal image and maximum RoI can widely capture individual muscle fascicles, and the change in EI would occur through an increase in the amplitude of the reflective wave at the muscle fascicle. Furthermore, we assessed the relationship between EI and pennation angle to explore the reason for EI change with ankle angle change. We hypothesized that the increase in EI would be partially explained by the increase in the pennation angle.

## 2 Materials and methods

### 2.1 Participants

A total of 16 healthy young male volunteers (age, 24.1 ± 2.8 years; height, 172.9 ± 5.2 cm; mass, 66.9 ± 9.0 kg) who had no history of ankle and/or posterior lower leg injuries (e.g., the gastrocnemius, soleus, or Achilles tendon) participated in this study. The sample size was calculated using G*Power 3.1 (Heinrich Heine University, Düsseldorf, Germany). The calculation was performed with an alpha level of 0.05, a power of 0.8, and an effect size of 0.4 [large] (Cohen, 2013), resulting in 16 participants. All the participants provided written informed consent prior to the experiments. The study was approved by our institutional ethics committee (R0233-3).

### 2.2 Protocols

The participants were asked to lay prone with their knee fully extended on the dynamometer (Biodex system 4.0; Biodex Medical Systems Inc., Shirley, New York, United States). The right foot was fixed on the footplate with the center of the right ankle joint aligned with the center of the dynamometer’s shaft, and then the trunk, pelvis, and right thigh were fixed with the attached belts.

**FIGURE 1:**
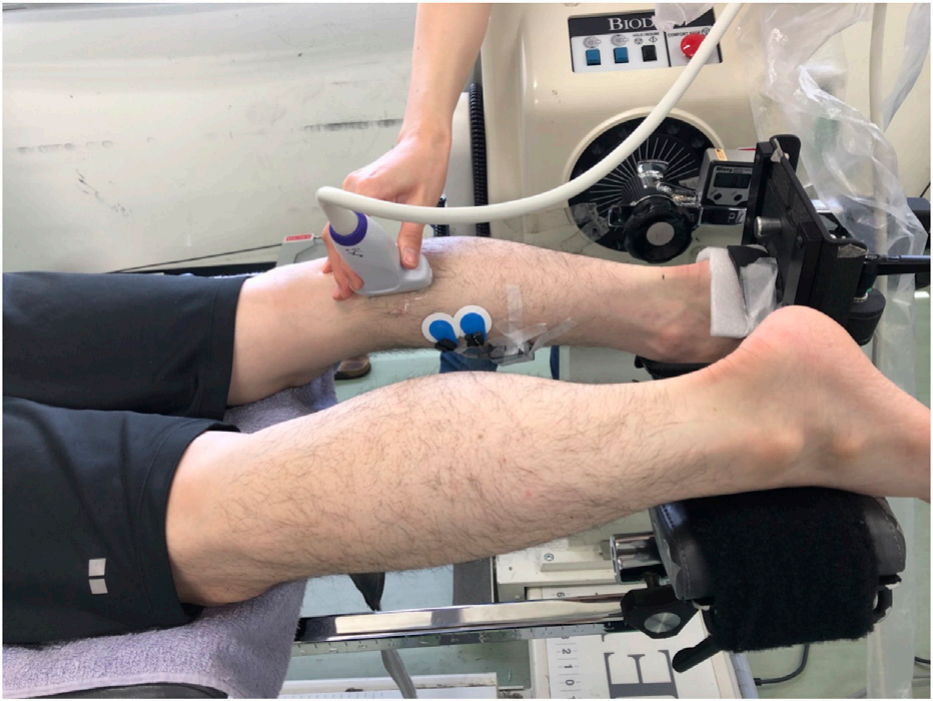
Measurement settings for shear modulus, echo intensity, and pennation angle.

The shear modulus, EI, and pennation angle of the right medial gastrocnemius muscle (MG) were measured at 20° and 10° of ankle plantar-flexion (PF20 and PF10, respectively), and at 0° (neutral position: NP), 10° and 20° of ankle dorsiflexion (DF10 and DF20, respectively) using the ultrasonography system (Aixplorer version 12.2.0; SuperSonic Imagine, Aix-en-Provence, France) (Figure 1). Measurement order of the angles of the ankle was randomly determined for each participant using the RAND function of Microsoft Excel (Microsoft Japan Co., Ltd., Tokyo, Japan). First, the shear modulus was measured at five different angles of the ankle, and then, the longitudinal B-mode images were scanned at the same ankle angle order as followed for the shear modulus, which was then followed by the scanning of the transverse B-mode images. The same examiner scanned each angle twice for all participants. To avoid the stretching effects, the two scans were performed within 1 min after setting the ankle angle, because a previous study showed that muscle stiffness did not change after 1 min stretching (Nakamura et al., 2013). If it took more than 1 min, the scan was performed again after a rest with the ankle in 20° plantar-flexed position. About 30 s of rest at 20° plantar-flexed position was set between each ankle angle.

The measurement was performed at 5 cm medial from the proximal 30% point of the line from the center of the popliteal crest to the lateral malleolus, as referring to previous studies (Nakamura et al., 2014; Saeki et al., 2017). If the vessels or the lateral gastrocnemius muscle were exposed, the measurement was performed by adding 1 cm more to the medial direction. The same measurement site was used for all the angles by marking where the initial ultrasound probe was set up on the skin.

Muscle activity of the MG during the ultrasound measurements was assessed using surface electromyography (Telemyo DTS, Noraxon United States, Inc., Scottsdale AZ, United States). To normalize electromyographic data, the muscle activity during maximal voluntary contraction was measured after the ultrasound measurements.

### 2.3 Shear modulus

Shear modulus of MG was measured by the ultrasound shear wave elastography (Aixplorer) with the probe of SL 15–4 (4–15 MHZ, linear-array, 50-mm wide; Supersonic Imagine, Aix-en-Provence, France). The shear wave elastography was set with the following parameters: 1) mode: musculoskeletal (MSK)/muscle mode; 2) opt: penetration mode; 3) persistence: medium; 4) smoothing: 5. Measurement was performed when the probe was applied perpendicularly to the muscle fascicles of MG, such that the muscle fascicles and fascia were displayed as clear as possible. The following analysis was performed by another examiner: the average shear wave group velocity (Vs) was calculated by using a 12–15 mm diameter circular Q-box, which was set up on the muscle belly avoiding the fascia and was as large as possible. This image analysis was performed once for each image. Shear modulus (G) was calculated using the following formula, with the muscle tissue density *ρ* [kg/m^3^] set to 1,000 kg/m^3^ (Gennisson et al., 2010):

$$G\;\left[\text{kPa}\right]=\rho Vs^{2}$$

In previous studies, this method has been demonstrated to exhibit good reliability for the measurement of shear modulus (Nakamura et al., 2014; Saeki et al., 2017).

### 2.4 Echo intensity and pennation angle

**FIGURE 2:**
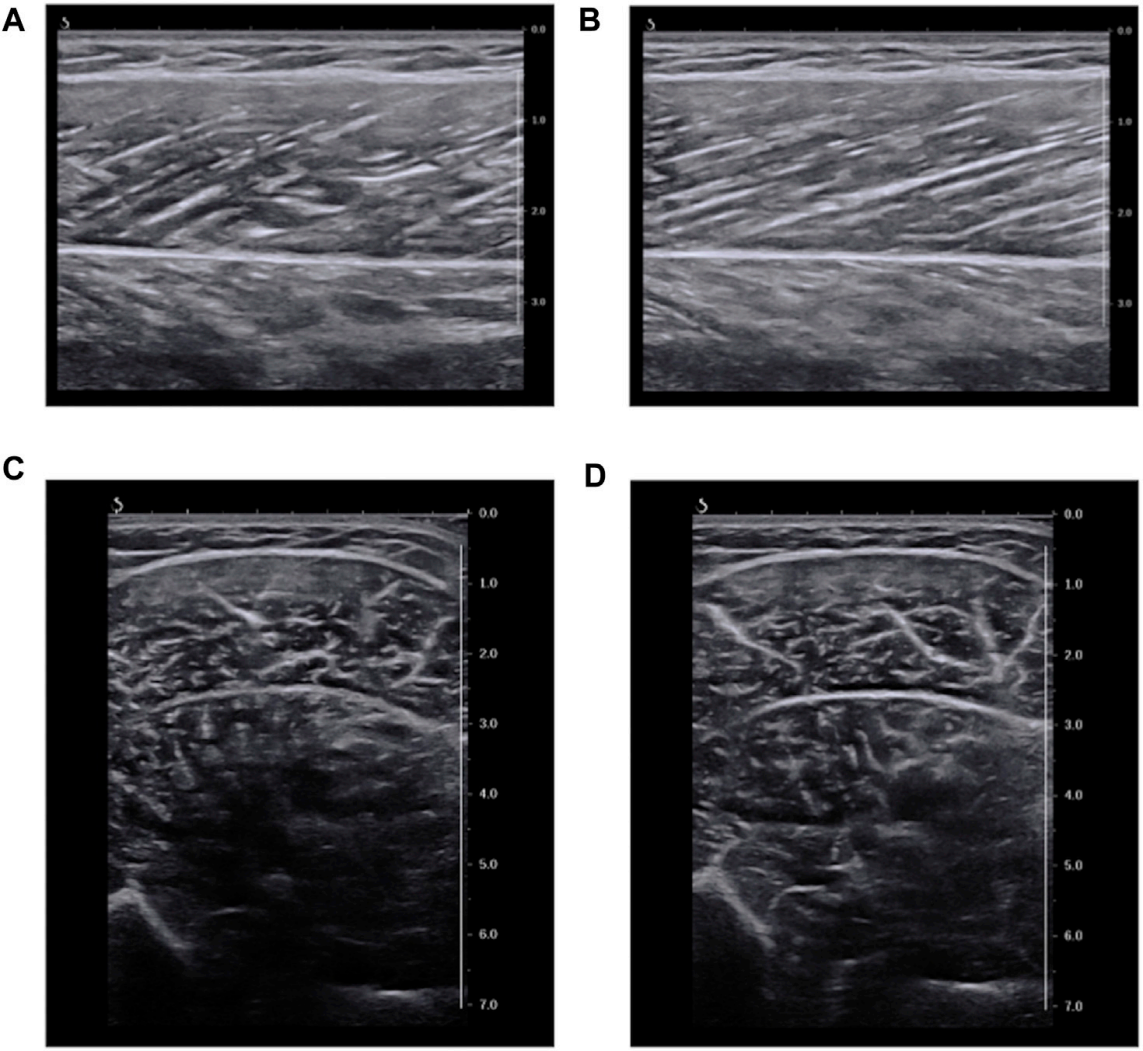
Representative ultrasound B-mode image. **(A)**: longitudinal images at 20° ankle plantar-flexion. **(B)**: longitudinal images at 20° ankle dorsiflexion. **(C)**: transverse images at 20° ankle plantar-flexion. **(D)**: transverse images at 20° ankle dorsiflexion. The values of echo intensity calculated using a region of interest (RoI) which included as much of the muscle as possible while avoiding fasciae and bone (maximum RoI) were **(A)** 109.3 a.u., **(B)** 118.4 a.u., **(C)** 76.0 a.u., and **(D)** 77.8 a.u.

To evaluate EI, B-mode images of MG were scanned longitudinally and transversely to the muscle fascicles using the ultrasonography (Aixplorer) with the probe of SL15–4 (Figure 2). The longitudinally scanned images were also used to evaluate the pennation angle. The scan settings for capturing the longitudinal images were: depth of 4.0 cm; focus of 0.5–3.0 cm; and gain of 58%. The images were taken when the muscle fascicles and fascia were visible as clear as possible. For capturing the transverse images, depth of 7.0 cm, focus of 0.5–7.0 cm and gain of 58% were used, and the scan was performed when the bone and fascia were visible as clear as possible.

**FIGURE 3:**
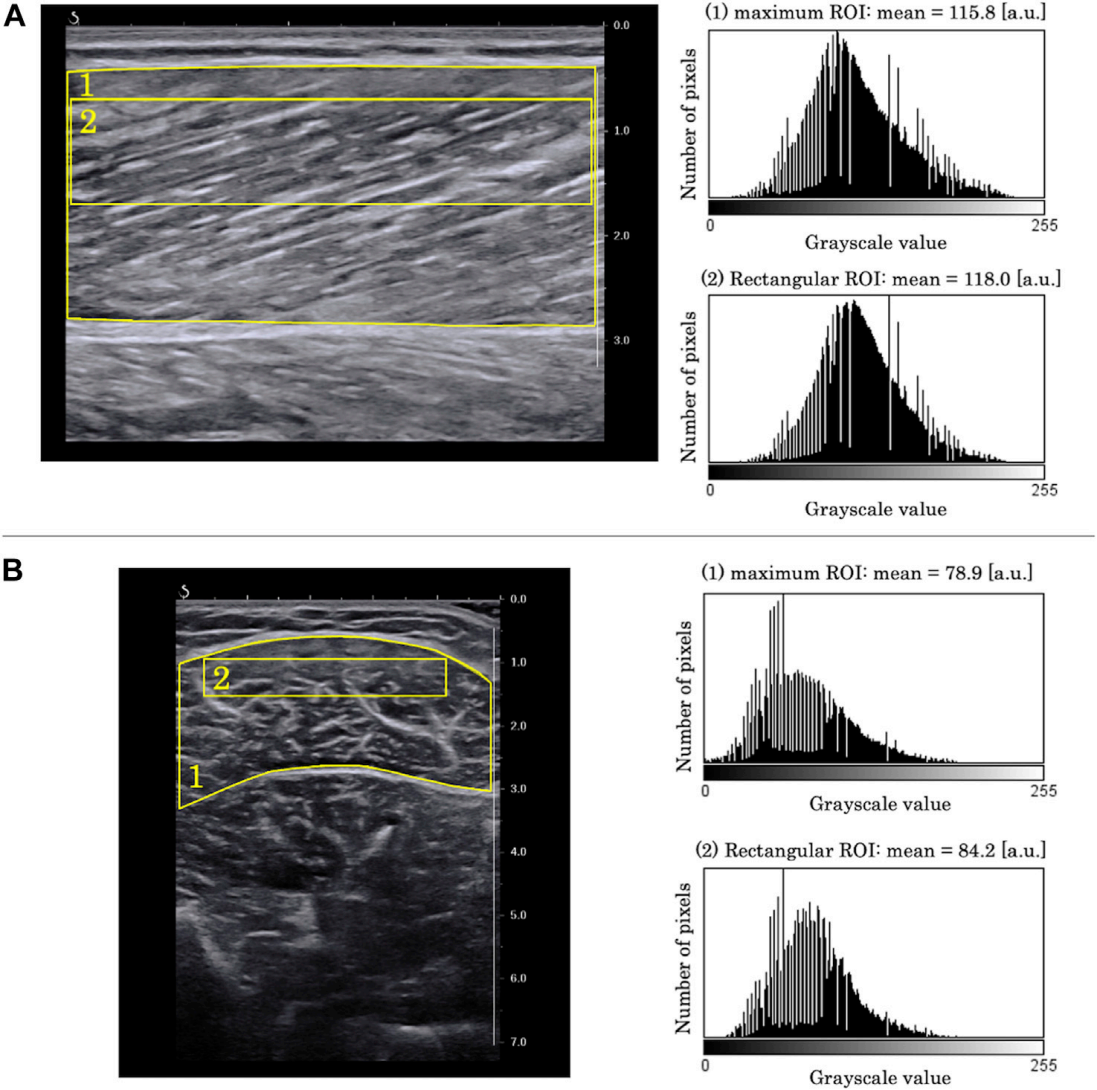
Examples of grayscale analysis in **(A)** longitudinal B-mode image and **(B)** transverse B-mode image. **(1)** a region of interest (RoI) which included as much of the muscle as possible while avoiding fasciae and bone (maximum RoI). **(2)** a region of interest whose size and depth were identical for all images of all participants (rectangular RoI). Grayscale histograms generated from each RoI are presented in the right panels.

The EI and pennation angle were quantified using the ImageJ software (National Institutes of Health, Bethesda, Maryland, United States) by another examiner than the one who scanned the B-mode images. The EI was determined as the average of grayscale values of the RoI set in each B-mode image, which ranged from 0 [white] to 255 [black]. Grayscale analysis was performed using the two RoIs for each B-mode image: one which included as much of the muscle as possible while avoiding fasciae and bone (maximum RoI), and a rectangular one whose size and depth were identical for all the images captured from all the participants (rectangular RoI) (Caresio et al., 2015). The rectangular RoI was assessed to minimize the effect of changes in muscle thickness and subcutaneous adipose tissue thickness on EI (Pinto et al., 2023). The representative longitudinal and transverse images are shown in Figure 3. In this study, the rectangular RoI for longitudinal image was set at a height of 1 cm (0.7–1.7 cm depth) and a width of 5 cm. For the transverse image, the rectangular RoI was set at a height of 0.6 cm (0.9–1.5 cm depth) and a width of 3.8 cm. This image analysis was performed once for each RoI and each image.

The pennation angle was determined as the angle between the deep aponeurosis and the muscle fascicle (Inami et al., 2017; Peng et al., 2017). Image analysis was performed three times per image using ImageJ, and the average value was calculated as the pennation angle for the image.

### 2.5 Electromyography

The muscle activity of the right MG during the ultrasound measurements was assessed using surface electromyography at 1,500 Hz. The electrodes were attached distally and medially from the ultrasound measurement point so as not to interfere with the probe (Figure 1). The muscle activity during the maximal voluntary ankle plantar-flexion was measured at 80° of hip flexion, and with the full knee extension at 0° of ankle plantar- or dorsi-flexion after the ultrasound measurements. The activity of MG was recorded for 3 s twice, with a 1 min rest between the two trials. The 50 ms root mean square was calculated after the rectification. The MG activity during the ultrasound measurements was determined as the normalized value of the maximum values obtained during the two maximal ankle plantar-flexion trials. The EMG activity of the MG was 1.5% ± 1.2% of the MVC during all ultrasound measurements.

### 2.6 Statistical analysis

To test the intra-rater reliability of the measurement of EI and pennation angle in advance, the intraclass correlation coefficients (ICCs) were calculated using the preliminary data collected from the five healthy male volunteers (age, 26.0 ± 3.9 years; height, 171.8 ± 5.0 cm; mass, 67.8 ± 11.8 kg). Longitudinal and transverse B-mode images were scanned three times each at 20° and 10° ankle plantar-flexion, and at 0°, 10° and 20° ankle dorsiflexion, as described above. The EI was quantified using the maximum RoIs and rectangular RoIs of each image. The ICC(1,1) values for EI of longitudinal and transverse images that were assessed using the maximum RoIs and rectangular RoIs were calculated using the three measured values of the five volunteers, respectively. The ICC(1,1) values for the EI of longitudinal images at each angle that were assessed using maximum RoI and rectangular RoI were recorded as 0.872–0.950 and 0.831–0.943, respectively. For the EI of transverse images, the ICC(1,1) values were recorded as 0.754–0.986 for maximum RoI, and 0.754–0.983 for rectangular RoI (Table 1). The ICC (1,1) value for the pennation angle was also calculated using the longitudinal B-mode images at 0° of ankle dorsiflexion from the same dataset, resulting in 0.695. According to a previous study (Landis and Koch, 1977), these ICC values are indicative of substantial reliability.

**TABLE 1:** The ICC(1,1) values of the echo intensity.

Ankle angle	Echo intensity of longitudinal images		Echo intensity of transverse images	
	Maximum RoI	Rectangular RoI	Maximum RoI	Rectangular RoI
20° plantar-flexion	0.934	0.931	0.969	0.960
10° plantar-flexion	0.872	0.831	0.986	0.943
0° plantar-/dorsiflexion	0.920	0.929	0.956	0.983
10° dorsiflexion	0.925	0.943	0.867	0.754
20° dorsiflexion	0.950	0.918	0.754	0.926

ICC, interclass correlation coefficient. Maximum RoI, region of interest that included as much of the muscle as possible while avoiding fasciae and bone. Rectangular RoI, region of interest for which the size and depth were identical for all images captured from all the participants.

The normality of shear modulus, EI, and pennation angle data at each angle was confirmed using the Shapiro-Wilk test, and the data was not significantly different from a normal distribution (*p* > 0.05). Using the ankle angle as a factor, the repeated measures analysis of variance (ANOVA) was used to evaluate the changes in shear modulus, EI, and pennation angle of the MG with ankle angle change. When repeated measures ANOVA showed a main effect of angle, the Bonferroni test was performed as a *post hoc* analysis. The average value of two images scanned at each angle was used for the repeated measures ANOVA.

To show the relationship between shear modulus of MG and passive ankle dorsiflexion, the Pearson’s correlation coefficients (*r*) were calculated between the shear modulus and ankle angle individually for each participant. Similarly, the Pearson’s correlation coefficients (*r*) between EI and ankle angle were calculated individually for each participant in order to investigate whether the changes in EI are correlated with the passive ankle dorsiflexion. For correlation analysis, ankle plantar-flexion angle was expressed using “−” and dorsiflexion was expressed using “+”, which means that the ankle angles of 20° and 10° plantar-flexion, and 0°, 10° and 20° dorsiflexion were represented as −20° and −10°, and 0°, +10° and +20°, respectively. The results are presented as average ± standard deviation (SD) of *r*-values obtained for the 16 participants. In addition, distributions of *r*-values are demonstrated using the boxplots.

Furthermore, the relationship between shear modulus and EI was examined by calculating the Pearson’s correlation coefficient (*r*) for each participant. For this analysis, the average of two images scanned at each angle was determined as the value at each angle. To investigate the change in EI with the change in pennation angle, Pearson’s correlation analysis was performed for each participant. These results are presented as average ± SD of the *r*-values obtained for the 16 participants. SPSS software (version 22.0; SPSS Japan Inc., Tokyo, Japan) was used for all statistical analyses. The significance level was set at 0.05.

## 3 Results

The shear modulus, EI, and pennation angle at each ankle angle are presented in Table 2. For shear modulus, the repeated measures ANOVA showed a significant main effect of angle (*p* < 0.01, partial *η* ^2^ = 0.935), and *post hoc* analysis showed significant changes between all angles except between PF20 and PF10 and between PF10 and NP. For the EI of longitudinal images, the repeated measures ANOVA showed a significant main effect of angle for both maximum and rectangular RoI (*p* < 0.01, partial *η* ^2^ = 0.623 and 0.515, respectively). The *post hoc* analysis for the maximum RoI showed a significant increase from PF20 to the other angles, from PF10 to DF10, and DF20, and from NP to DF20 for maximum RoI (*p* < 0.05). The results for the rectangular RoI showed a significant increase from PF20 to NP, DF10 and DF20, from PF10 to DF10 and DF20, and from NP to DF20 (*p* < 0.05). With regard to the EI of the transverse images, there was a significant main effect of angle for the maximum and rectangular RoI (*p* < 0.05, partial *η* ^2^ = 0.220 and 0.213, respectively), and significant differences were shown only with DF20. For the pennation angle, the repeated measures ANOVA showed a significant main effect of angle (*p* < 0.01, partial *η* ^2^ = 0.754), and *post hoc* analysis showed significant changes between all angles except between NP and DF10 and between DF10 and DF20.

**TABLE 2:** Shear modulus [kPa], echo intensity [a.u.], and pennation angle [°] at each ankle angle.

	PF 20°	PF 10°	PF/DF 0° (NP)	DF 10°	DF 20°
**Shear modulus**	4.7 ± 1.1	6.8 ± 1.5	12.0 ± 2.4^a^	24.7 ± 5.8^a^ ^,^ ^b^ ^,^ ^c^	55.8 ± 13.7^a^ ^,^ ^b^ ^,^ ^c^ ^,^ ^d^
**Echo intensity of longitudinal images**					
Maximum RoI	108.3 ± 9.3	111.1 ± 8.5^a^	112.4 ± 8.6^a^	114.2 ± 9.3^a^ ^,^ ^b^	115.5 ± 9.9^a^ ^,^ ^b^ ^,^ ^c^
Rectangular RoI	107.2 ± 10.4	109.4 ± 9.7	110.3 ± 9.4^a^	112.8 ± 10.2^a^ ^,^ ^b^	114.2 ± 10.4^a^ ^,^ ^b^ ^,^ ^c^
**Echo intensity of transverse images**					
Maximum RoI	68.3 ± 9.5	68.1 ± 9.2	67.9 ± 8.5	68.3 ± 8.5	70.4 ± 8.2^a^ ^,^ ^b^ ^,^ ^c^ ^,^ ^d^
Rectangular RoI	72.4 ± 11.1	70.9 ± 10.3	72.8 ± 9.8	73.0 ± 10.1	75.1 ± 9.9^b^
**Pennation angle**	22.2 ± 2.0	20.3 ± 1.7^a^	18.8 ± 2.0^a^ ^,^ ^b^	17.5 ± 2.0^a^ ^,^ ^b^	16.5 ± 1.5^a^ ^,^ ^b^ ^,^ ^c^

Values are expressed as mean ± standard deviation. PF, plantar-flexion; DF, dorsiflexion; NP, neutral position. Maximum RoI, region of interest that included as much of the muscle as possible while avoiding fasciae and bone. Rectangular RoI, region of interest for which the size and depth were identical for all the images captured from all the participants.

^a^ There was a significant change from the value at PF20 (*p* < 0.05).

^b^ There was a significant change from the value at PF10 (*p* < 0.05).

^c^ There was a significant change from the value at NP (*p* < 0.05).

^d^ There was a significant change from the value at DF10 (*p* < 0.05).

**FIGURE 4:**
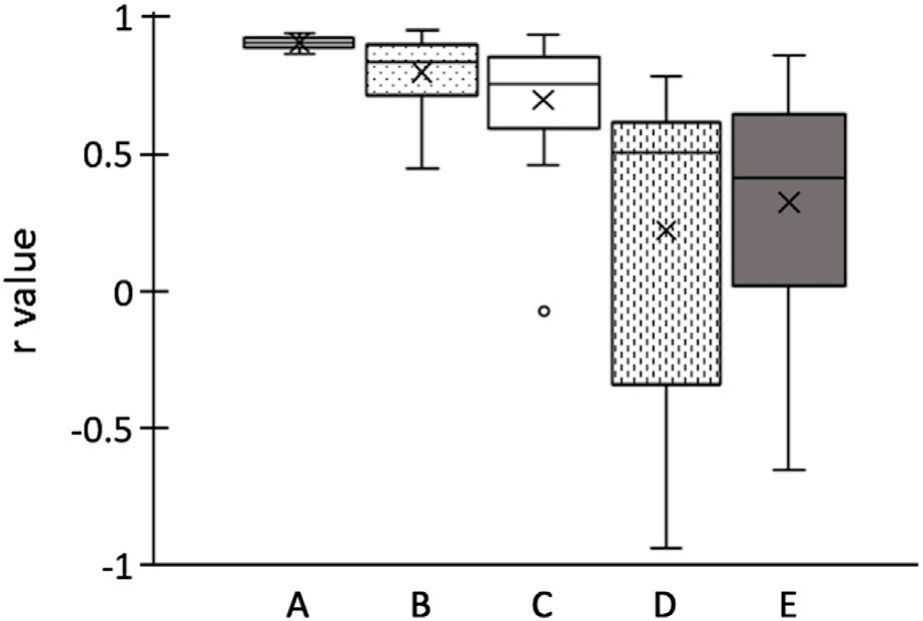
Boxplots of Pearson’s correlation coefficients *r* of ankle angle for each participant. **(A)** shear elastic modulus; echo intensity of longitudinal images calculated using **(B)** maximum RoI and **(C)** rectangular RoI; echo intensity of transverse images calculated using **(D)** maximum RoI and **(E)** rectangular RoI. Boxplots embrace 25th and 75th percentiles with a horizontal line drawn in the middle to show the median. Whiskers show the maximum and minimum variability outside the upper and lower quartiles. Outliers are presented with dots.

In Figure 4, the boxplots depicting the Pearson’s correlation coefficients between the shear modulus or EI and the ankle angle for each participant are presented. Average (±SD) Pearson’s correlation coefficient between the shear modulus and the ankle angle for each participant was calculated as 0.904 ± 0.021, which is indicative of a strong positive correlation. The Pearson’s correlation coefficients between the EI of longitudinal images and the ankle angle were 0.797 ± 0.137 for the maximum RoI, and 0.698 ± 0.249 for the rectangular RoI, which also indicates towards relatively strong positive correlations. The Pearson’s correlation coefficients between the EI of transverse images and the ankle angle were 0.222 ± 0.583 for the maximum RoI, and 0.323 ± 0.439 for the rectangular RoI, which indicates towards weak positive correlations and is attributed to the large SDs.

The average (±SD) Pearson’s correlation coefficient between the shear modulus and the EI of longitudinal images for each participant was calculated as 0.684 ± 0.270 for the maximum RoI, and 0.611 ± 0.342 for the rectangular RoI. As for the EI of the transverse images, the Pearson’s correlation coefficient of the shear modulus was 0.514 ± 0.603 for the maximum RoI and 0.409 ± 0.471 for the rectangular RoI.

As for the relationship between the EI and pennation angle, the average (±SD) Pearson’s correlation coefficient between the pennation angle and the EI of the longitudinal image was −0.669 ± 0.179 for the maximum RoI and −0.581 ± 0.256 for the rectangular RoI. The correlations between the pennation angle and the EI of the transverse images was −0.264 ± 0.572 for the maximum RoI and −0.204 ± 0.418 for the rectangular RoI.

## 4 Discussion

In this study, we investigated the changes in shear modulus and EI of MG with muscle elongation by passive ankle dorsiflexion. To the best of our knowledge, this is the first study investigating whether the muscle EI can be used as an indicator of muscle elongation. Shear modulus and EI of longitudinal images were increased with ankle dorsiflexion and positively correlated with ankle angle, while the correlation of EI was not as strong as that of shear modulus. These results indicate that the two potential indicators increased with passive ankle dorsiflexion, which supported our hypothesis as well as the results of a previous study performed with regard to the shear modulus (Hug et al., 2013). While assessing the EI of longitudinal images, the *r*-values with ankle angle were higher when the calculations were performed using the maximum RoI than that of the rectangular RoI. This indicates that maximum RoI is more recommended than rectangular RoI to assess the changes in EI of MG with changes in ankle angle. Furthermore, there were positive relationships between the shear modulus and EI of the longitudinal images for both maximum and rectangular RoIs, reinforcing the hypothesis that the EI of the longitudinal images increases with muscle elongation. The negative relationship between EI and pennation angle indicates that the increase in EI in the longitudinal images was partially explained by a decrease in the pennation angle. On the contrary, the EI of the transverse images increased only at DF20 compared with other angles, and weak positive correlations with high variability among participants were observed between the ankle angle and EI that were calculated for the transverse images. In addition, the relationships of shear modulus or pennation angle were weaker in the EI of the transverse images than the EI of the longitudinal images. These findings indicate that a small change in the EI of transverse images occurs with muscle elongation, and there is little relationship with the change in the pennation angle.

The results showed that the *r*-values of ankle angle and shear modulus were higher for the EIs of the longitudinal images than those of the transverse images, which suggests that using longitudinal B-mode images would be more suitable for assessing the changes in EI with muscle elongation by passive ankle dorsiflexion. The results of correlation analysis showed that the pennation angle was negatively correlated with the EI, and the absolute value of the correlation coefficients |*r*| were higher for the EI of the longitudinal images (average |*r*| > 0.5) than the transverse images (average |*r|* < 0.3). This suggests that the decrease in the pennation angle could partially explain the increase in EI, particularly of the longitudinal images. Longitudinal B-mode images, which take into consideration a broader range of muscle fascicles than transverse images, may be more suitable for capturing the following possible muscle fascicle changes with ankle dorsiflexion. First, changes in the pennation angle may be responsible for the change in the EI of the longitudinal images with a change in the joint angle. When the pennation angle of MG decreased with the ankle dorsiflexed (Abellaneda et al., 2009), the muscle fascicles became perpendicular to the ultrasound waves such that the reflection of the ultrasound waves increased in the perimysium surrounding the muscle fascicle, which further resulted in a higher EI. Second, the increase in perimysium stretching may have contributed to the increase in EI with ankle dorsiflexion. B-mode signal intensity, which is known as EI, had previously been shown to increase with skin stretching (Pan et al., 1998). The authors of that study considered that this change in B-mode signal intensity could be associated with the decrease in ultrasound attenuation coefficient resulting from reorientation of the collagen matrix with skin stretching. Considering the perimysium is also composed of collagen fibers, the increase in muscle EI with ankle dorsiflexion might be attributed partially to an increase in the reflection of ultrasound waves upon stretching the perimysium. Third, changes in the EI in the longitudinal images may also have been affected by changes in the number or range of displayed muscle fascicles in the B-mode image as a result of the three-dimensional rearrangement of muscle fascicles (Takahashi et al., 2023). This three-dimensional rearrangement may allow muscle fascicles that are out of the B-mode image in the slacked position to be captured in the lengthened position. This indicates an increase in the reflective planes of the ultrasound wave, which results in an increase in the EI. However, the reason why the EI changes in the longitudinal and transverse images were different remains unclear from the present study, except for that of the effect of the pennation angle. Therefore, further studies are needed to investigate whether the EI increases in other pennate muscles.

**FIGURE 5:**
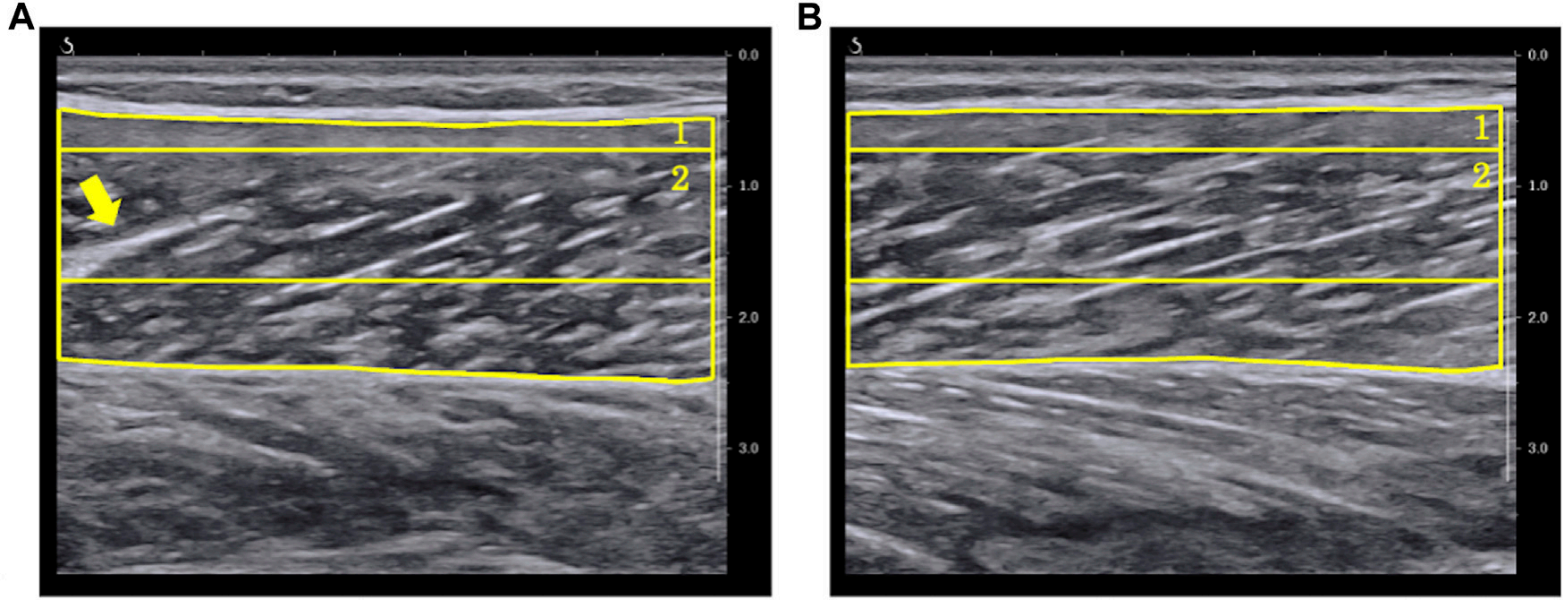
Ultrasound B-mode images with the ankle in **(A)** 20° plantar-flexion and **(B)** 20° dorsiflexion for the participant who showed minimum *r*-value between echo intensity and ankle angle. In the image at 20° ankle plantar-flexion, the arrow signifies a fiber (displayed in white) that presents like an intramuscular tendon. **(1)** A region of interest (RoI) which included as much of the muscle as possible while avoiding fasciae and bone (maximum RoI). **(2)** A region of interest whose size and depth were identical for all images of all participants (rectangular RoI).

In the present study, we also investigated whether the changes in EI with the change in ankle joint differed depending on the RoI setting by analyzing EI using the two previously reported RoIs (Caresio et al., 2015). In the longitudinal images, the *r*-values of EI calculated using maximum RoI showed higher average and smaller SD than that of the rectangular RoI. Using the maximum RoI may induce higher EI values due to the decrease in ultrasound attenuation when the muscle thickness decreases with a change in joint angle (Varanoske et al., 2021). On the other hand, the maximum RoI has the advantage of capturing changes in a larger range of muscle architecture. In this study, while the minimum *r*-value was recorded as 0.448 for the maximum RoI, the minimum *r*-value for the rectangular RoI was an outlier and was recorded as −0.076 (Figure 4). As presented in Figure 5, the ultrasound B-mode images of the participant who exhibited the minimum *r*-values for both RoIs displayed white regions that appeared as noncontractile elements. These regions were captured specifically during ankle plantar-flexion and were not present in the images captured during ankle dorsiflexion. Noncontractile elements, such as fibrous tissue or intramuscular adipose tissue, affect EI values (Pillen et al., 2009; Arts et al., 2012; Young et al., 2015). Thus, while using the rectangular RoI (which is smaller than the maximum RoI), artifacts might affect the assessment of the change in EI and could prevent the capture of the actual change, resulting in weak correlation coefficients. Therefore, analyzing B-mode images using the maximum RoI could be more recommended and reliable than the rectangular RoI to assess the changes in EI of MG with ankle angle change. However, the rectangular RoI could be more appropriate than the maximum RoI, if the analytical range is altered prominently due to a high variation in the thickness of the muscle or subcutaneous tissue among the angles of the joint or the participants. Further research is required to investigate which RoIs are appropriate for other muscles.

There are certain limitations in the present study. First, in this study, we assessed only MG to investigate the changes in muscle EI with muscle elongation. Therefore, further research is needed to clarify the possibility of using EI as an indicator of muscle elongation, which could be performed by investigating whether EI also changes with muscle elongation in fusiform muscles similar to that in pennate muscle as demonstrated in the current study. Second, only healthy young males were chosen to participate in this study. In previous studies, it has been reported that EI was higher in older adults and female participants (Caresio et al., 2015). Yoshiko et al. (2023) reported a relationship between the amount of intramuscular adipose tissue and the change in shear wave velocity or the change in fascicle length. In female or older individuals who have high muscle EI and more intramuscular adipose tissue, the changes in muscle architecture with muscle elongation may differ from the present study, resulting in different changes in EI with muscle elongation. Therefore, it is important to investigate the changes in EI with joint angle changes in the participants who exhibit high EI at the baseline.

## 5 Conclusion

This study investigated the changes in shear modulus and EI of MG with muscle elongation by passive ankle dorsiflexion. The results of this study revealed that the shear modulus and EI of longitudinal images, and not of transverse images increased when the ankle was dorsiflexed, which means when MG was stretched. Regarding the RoI settings, the results of this study indicated that the maximum RoI, which included as much of the muscle as possible, could be more recommended and reliable to use for calculating EI as compare to the rectangular RoI, in which the size and depth were identical for all images. While the correlations of EIs with the ankle angle were not as strong as shear modulus’s, EI of longitudinal images calculated using maximum RoI might be useful for quantifying the muscle elongation in the clinics because of its comparatively low cost.

## Data Availability

The raw data supporting the conclusion of this article will be made available by the authors, without undue reservation.
